# Legacy 1

**DOI:** 10.1007/s10912-016-9417-1

**Published:** 2016-12-03

**Authors:** Tess Hurson-Maginess

**Affiliations:** School of Education, Queen’s University, Belfast, UK

So now I have

a roomy lilac cardigan

But not the missing twin

Which had made the set

I chivvied her to wear,

And she saw for a moment

Her own hieratic dark grey eyes

Set off to just perfection

by that soft mauve wool.

And a stylish duckegg plate I brought

From one of my adventures—

Free and full of guff and gravitas

And mortgaged to the hilt

Of her gritted, contending heart—

The plate for hanging up

She trussed with a ragged strip

Of worn out sheet before consignment

To a drawer bottomed with curtain hooks,

medals, florins, a calendar page

In a cursive, rushing hand, noting the birthdates

Breed and sex of four dropped calves,

safety pins, curlers, large glass spectacles.

And a pretty little wicker chair

She bought in a fit of good taste

I had damn all to do with.

A thing I did not shape or predicate,

In all our battles, no more than she.

Warriors she called us towards the end,

Tightening us to occasionally cherish

Some lonely impulse of delight

Among the trumphery of love.

30, 31 April 2010

